# Autonomous Integrated Navigation for Indoor Robots Utilizing On-Line Iterated Extended Rauch-Tung-Striebel Smoothing

**DOI:** 10.3390/s131215937

**Published:** 2013-11-25

**Authors:** Yuan Xu, Xiyuan Chen, Qinghua Li

**Affiliations:** Key Laboratory of Micro-Inertial Instrument and Advanced Navigation Technology, Ministry of Education, School of Instrument Science and Engineering, Southeast University, Nanjing 210096, China; E-Mails: xy_abric@126.com (Y.X.); liqinghua1977@163.com (Q.L.)

**Keywords:** inertial navigation systems (INS), integrated navigation, iterated extended Kalman filter (IEKF), extended Rauch-Tung-Striebel smoother (ERTSS), average filtering

## Abstract

In order to reduce the estimated errors of the inertial navigation system (INS)/Wireless sensor network (WSN)-integrated navigation for mobile robots indoors, this work proposes an on-line iterated extended Rauch-Tung-Striebel smoothing (IERTSS) utilizing inertial measuring units (IMUs) and an ultrasonic positioning system. In this mode, an iterated Extended Kalman filter (IEKF) is used in forward data processing of the Extended Rauch-Tung-Striebel smoothing (ERTSS) to improve the accuracy of the filtering output for the smoother. Furthermore, in order to achieve the on-line smoothing, IERTSS is embedded into the average filter. For verification, a real indoor test has been done to assess the performance of the proposed method. The results show that the proposed method is effective in reducing the errors compared with the conventional schemes.

## 1. Introduction

Autonomous mobile robots have increasingly been used in a wide range of applications [1]. One key issue for mobile robots is the ability to obtain their navigation information (such as position, velocity and so on). In order to obtain accurate navigation information indoors, a number of methods for localization with various sensors and different degrees of precision were proposed over the past few decades [1–3].

The main research approaches for indoor localization include beacon-based solutions and beacon-free solutions [4]. Beacon-based solutions employ reference nodes (RNs) with known location to complete the localization of blind nodes (BNs). Its principle is similar to that of global positioning systems (GPS), but the communication technology used is short-range radio, such as WiFi, UWB, RFID, ZigBee, ultrasound, *etc.* Many researchers have attempted to employ beacon-based solutions for indoor localization. For instance, Shirehjini *et al.* proposed an RFID-based position and orientation measurement system for mobile objects in [5]; Park *et al.* employed ZigBee for an indoor location system [6]. Most of the abovementioned attempts employ the measurement of one or several physical parameters of the radio signal transmitted between the RNs and BNs to complete the wireless localization. Due to the influence of the building structures in indoor environments, the accuracy is about one meter. In order to obtain higher accuracy, some researchers employ ultrasonic waves, and the time of arrival (TOA) mode to complete the distance measurement. For instance, Minami *et al.* proposed a fully distributed localization system based on ultrasound, where the localization accuracy was about 20 cm with 24 devices [7], and Saad *et al.* proposed high-accuracy reference-free ultrasonic location estimation in [2]. Ultrasonic sensors have been shown to be a simple but powerful system for this mode, however, it needs has a high density of RNs to maintain the localization accuracy, which is not practical for large localization areas. Differing from beacon-based solutions relying on RNs, beacon-free ones are a self-contained system capable of providing positioning information independently [8]. Some attempts using inertial navigation system (INS)-based beacon-free solutions have been used in integrated outdoor navigation [9]. For example, a GPS/INS navigation system for launchers and re-entry vehicles was described by Boulade *et al.* in [10], and Xu *et al.* proposed a novel hybrid of least squares support vector machine (LS-SVM) and Kalman filter for GPS/INS integration in [11]. Like the integration navigation mentioned above, several approaches also employ INS-based beacon-free solutions for indoor navigation. For example, Ruiz *et al.* employed inertial measuring units (IMUs)/radio frequency identification (RFID) integrated navigation for pedestrian indoor navigation in [4]. Evennou *et al.* proposed a WiFi/INS integration navigation system for indoor mobile positioning in [12]. However, it should be pointed out that beacon-free solutions are poor in long-term self-contained navigation since the accuracy deteriorates with time [13,14], thus it is just a short-term compensation and therefore, it is not suitable for the precision control of indoor mobile robots.

In the data fusion for the integrated navigation system, the integration filter plays an important role in the navigation accuracy. One of the most popular information fusion algorithms is the Kalman filter (KF). However, its optimality heavily depends on linearity [15]. In order to overcome this problem, the extended KF (EKF) is used [14], but the linearization of a nonlinear system by Taylor series expansion and neglection of the truncated high-order terms will introduce a truncation error [15]. Then, the unscented KF (UKF) and iterated Extended Kalman filter (IEKF) are proposed. Although the UKF is able to overcome the shortcomings of the EKF, it needs more time to compute large numbers of samples [16]. The IEKF is able to reduce the bias and the estimation errors by adding only a few simple iterative operations. In order to obtain high accuracy, smoothing algorithms have been effectively applied for integrated navigation systems [17]. The Rauch-Tung-Striebel smoothing (RTSS) is one of the most popular methods [18]. RTSS was first proposed in [19], and it includes one forward data processing and one backward data processing step. Due to its robustness and effectiveness, it is widely used in navigation applications. However, it is only suitable for linear systems. In order to overcome this problem, some researchers employ the so-called Extended RTSS (ERTSS). In this mode, the forward data processing of ERTSS is implemented by EKF instead of KF, such as in [20]. In order to reduce the estimated error of the INS/WSN navigation indoors for mobile robots, this work proposes the design and implementation of an on-line iterated ERTSS (IERTSS). The IEKF is used to improve the filtering output accuracy of the ERTSS algorithm. Then, in order to achieve the on-line smoothing, the IERTSS is used in an average filter to smooth the errors of the INS during the output period. A real indoor test is used to evaluate the performance of the proposed method. The remainder of the paper is organized as follows: Section 2 gives the IERTSS embedded average filter design. The unbiased tightly-coupled integrated model for mobile robot navigation indoors is illustrated in Section 3. Section 4 gives the real indoor tests and performance. Finally, conclusions are given in Section 5.

## 2. On-Line Iterated Extended Rauch-Tung-Striebel Smoothing

In this section, a brief introduction to the IEKF and ERTSS will be given, and then, an on-line IERTSS will be proposed.

### 2.1. Iterated Extended Kalman Filter

It is assumed that a discrete-time model of a nonlinear system is given by Equations (1) and (2):

(1) $$X_{k}=f\left(X_{k-1}\right)+B_{k-1}\omega_{k-1}$$

(2) $$y_{k}=h\left({\text{X}}_{k}\right)+\upsilon_{k}$$

where **X***_k_* is the state vector at time *k*, **f**(**X***_k_*) is the system nonlinear function, **B***_k_* is the process noise input matrix, **y***_k_* is the observation vector, and h(**X***_k_*) is the observation nonlinear function. **ω***_k_* is the process noise, and **υ***_k_* is the observation noise. It is assumed that **ω***_k_* and **υ***_k_* are independent zero-mean white Gaussian sequences with covariance **Q** and **R**, respectively. The IEKF used in this paper involves the following recursive relations [17,19]:

(3) $${\hat{X}}_{k|k-1}=A_{k-1}{\hat{X}}_{k-1|k-1}$$

(4) $$P_{k|k-1}=A_{k-1}P_{k-1}{A_{k-1}}^{T}+Q$$

where $A_{k}=\frac{\partial f\left({\hat{X}}_{k|k}\right)}{\partial {\hat{X}}_{k|k}}$. Compared with the EKF, the IEKF employs a few simple iterative operations to reduce the bias and the estimation error after getting **X̂***_k|k-_*_1_ in Equation (3) and **P***_k|k_*_-1_ in Equation (4). The corresponding recursive relations are:

(5) $${\hat{X}}_{k|k}^{1}={\hat{X}}_{k|k-1}$$

(6) $$P_{k|k}^{1}=P_{k|k-1}$$

(7) $$K_{k}^{n}=P_{k|k-1}H^{n}{\left({\hat{X}}_{k|k}^{n}\right)}^{T}{\left[H^{n}\left({\hat{X}}_{k|k}^{n}\right)P_{k|k-1}H^{n}{\left({\hat{X}}_{k|k}^{n}\right)}^{T}+R\right]}^{-1}$$

(8) $${\hat{X}}_{k|k}^{n+1}={\hat{X}}_{k|k}^{n}+K_{k}^{n}\left[y_{k}-h^{n}\left({\hat{X}}_{k|k}^{n}\right)-H^{n}\left({\hat{X}}_{k|k}^{n}\right)\times \left({\hat{X}}_{k|k-1}-{\hat{X}}_{k|k}^{n}\right)\right]$$

(9) $$P_{k|k}^{n}=\left[I-K_{k}^{n}H^{n}\left({\hat{X}}_{k|k}^{n}\right)\right]P_{k|k-1}{\left[I-K_{k}^{n}H^{n}\left({\hat{X}}_{k|k}^{n}\right)\right]}^{T}+K_{k}^{n}R{\left(K_{k}^{n}\right)}^{T}$$

here, $H^{n}\left({\hat{X}}_{k|k}^{n}\right)=\frac{\partial h\left({\hat{X}}_{k|k}^{n}\right)}{\partial {\hat{X}}_{k|k}^{n}}$, *n* is the number of iteration and *n*=1,2,…,*j*. Then:

(10) $${\hat{X}}_{k|k}={\hat{X}}_{k|k}^{j}$$

(11) $$P_{k|k}=P_{k|k}^{j}$$

### 2.2. Extended Rauch-Tung-Striebel Smoothing

Consider the nonlinear system given by Equations (1) and (2), the forward data processing of ERTSS is utilizing a set of equations as follows:

(12) $${\hat{X}}_{k|k-1}=A_{k-1}{\hat{X}}_{k-1|k-1}$$

(13) $$P_{k|k-1}=A_{k-1}P_{k-1}{A_{k-1}}^{T}+Q$$

(14) $$K_{k}=P_{k|k-1}H_{k}^{T}{\left[H_{k}P_{k|k-1}H_{k}^{T}+R\right]}^{-1}$$

(15) $${\hat{X}}_{k|k}={\hat{X}}_{k|k-1}+K_{k}\left[z_{k}-h\left({\hat{X}}_{k|k-1}\right)\right]$$

(16) $$P_{k|k}=\left[I-K_{k}H_{k}\right]P_{k|k-1}$$

where $A_{k}=\frac{\partial f\left({\hat{X}}_{k|k}\right)}{\partial {\hat{X}}_{k|k}}$, $H_{k}=\frac{\partial h\left({\hat{X}}_{k|k}\right)}{\partial {\hat{X}}_{k|k}}$. The backward data processing propagates the filtering outputs and achieves the smoothing results by using the R-T-S formulation. It is computed with the following equations:

(17) $$K_{k}^{S}=P_{k|k}A_{k}^{T}{\left(P_{k+1|k}\right)}^{-1}$$

(18) $${\hat{X}}_{k|k}^{S}={\hat{X}}_{k|k}+K_{k}^{S}\left[{\hat{X}}_{k+1|k+1}^{S}-{\hat{X}}_{k|k-1}\right]$$

(19) $$P_{k}^{S}=P_{k|k}+K_{k}^{S}\left(P_{k+1}^{S}-P_{k|k-1}\right){\left(K_{k}^{S}\right)}^{T}$$

where the superscript *S* denotes the smoothing, and the recursion [Equations (17)–(19)] is started from the filtering output at the final time.

### 2.3. On-Line Iterated Extended Rauch-Tung-Striebel Smoothing

**Figure 1.**
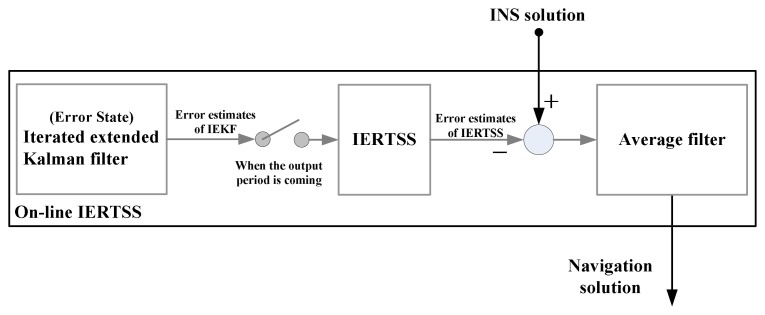
The flow chart of the on-line IERTSS.

As mentioned above, it can be seen that the filtering output accuracy of ERTSS is dependent on the EKF, however, the EKF will generate truncated errors since it employs Taylor series expansion to linearize the nonlinear system. In this work, in order to obtain a higher accuracy solution, the IEKF mentioned above is used in the forward data processing part of ERTSS (called IERTSS). Moreover, in order to achieve on-line smoothing, this work proposes an on-line IERTSS. The flow chart of this on-line IERTSS is shown in Figure 1. In this mode, the IEKF is used for optimal state estimation. When the output periods are coming, firstly, the IERTSS is employed to smooth the filtering output of IEKF between two data output periods. Then, the average value of the INS state estimation is computed with the INS solution and IERTSS solution.

## 3. Unbiased Tightly-Coupled Integrated Model for Mobile Robot Navigation Indoors

The unbiased tightly-coupled integrated model proposed in [21] is employed in this work. The continuous-time state equation of the filter is illustrated in Equation (20):

(20) $$\underset{{\dot{X}}_{k}}{\underset{︸}{\left[\begin{array}{c} \delta {\dot{P}}_{E,k}\\ \delta {\dot{V}}_{E,k}\\ \delta A\dot{c}c_{E,k}\\ \delta {\dot{P}}_{N,k}\\ \delta {\dot{V}}_{N,k}\\ \delta A\dot{c}c_{N,k} \end{array}\right]}}=\underset{A^{*}}{\underset{︸}{\left[\begin{array}{cccccc} 0 & 1 & T & 0 & 0 & 0\\ 0 & 0 & 1 & 0 & 0 & 0\\ 0 & 0 & 0 & 0 & 0 & 0\\ 0 & 0 & 0 & 0 & 1 & T\\ 0 & 0 & 0 & 0 & 0 & 1\\ 0 & 0 & 0 & 0 & 0 & 0 \end{array}\right]}}\underset{X_{k-1}}{\underset{︸}{\left[\begin{array}{c} \delta P_{E,k-1}\\ \delta V_{E,k-1}\\ \delta Acc_{E,k-1}\\ \delta P_{N,k-1}\\ \delta V_{N,k-1}\\ \delta Acc_{N,k-1} \end{array}\right]}}+W_{k}^{*}$$

where (*δP_E,K_*, *δP_N,K_*), (*δV_E,K_*, *δV_N,K_*) and (*δAcc_E,K_*, *δAcc_N,K_*) are the errors of position, velocity and acceleration measured by the INS in east and north direction at moment *k. T* is the sample time; $W_{k}^{*}$is the Gaussian process noise. Equation (20) can be transferred into a discrete-time state equation:

(21) $$\underset{X_{k}}{\underset{︸}{\left[\begin{array}{c} \delta P_{E,k}\\ \delta V_{E,k}\\ \delta Acc_{E,k}\\ \delta P_{N,k}\\ \delta V_{N,k}\\ \delta Acc_{N,k} \end{array}\right]}}=\underset{A}{\underset{︸}{\left[\begin{array}{cccccc} 1 & T & T^{2}/2 & 0 & 0 & 0\\ 0 & 1 & T & 0 & 0 & 0\\ 0 & 0 & 1 & 0 & 0 & 0\\ 0 & 0 & 0 & 1 & T & T^{2}/2\\ 0 & 0 & 0 & 0 & 1 & T\\ 0 & 0 & 0 & 0 & 0 & 1 \end{array}\right]}}\underset{X_{k-1}}{\underset{︸}{\left[\begin{array}{c} \delta P_{E,k-1}\\ \delta V_{E,k-1}\\ \delta Acc_{E,k-1}\\ \delta P_{N,k-1}\\ \delta V_{N,k-1}\\ \delta Acc_{N,k-1} \end{array}\right]}}+W_{k}$$

where **W***_k_* is the Gaussian process noise. Here, the position of the robot measured by the INS is denoted as $\left(P_{E}^{\text{INS}},P_{N}^{\text{INS}}\right)$, and the position of the RN is denoted as(*x,y*). Thus, the distance between the robot and the RN measured by the INS can be expressed as Equation (22):

(22) $$d_{i}^{\text{INS}}=\sqrt{{\left(P_{E}^{\text{INS}}-x_{i}\right)}^{2}+{\left(P_{N}^{\text{INS}}-y_{i}\right)}^{2}},i=1,2,\cdots ,m$$

where *m* is the number of the RN. Theoretically, the real distance between the robot and the RN is expressed as Equation (23):

(23) $$d_{i}^{\text{Real}}=\sqrt{{\left(P_{E}^{\text{Re}al}-x_{i}\right)}^{2}+{\left(P_{N}^{\text{Re}al}-y_{i}\right)}^{2}},i=1,2,\cdots ,m$$

where $d_{i}^{\text{Real}}$ is the real distance between the robot and the RN, $\left(P_{E}^{\text{Re}al},P_{N}^{\text{Re}al}\right)$ is the real position of the robot. The difference between ${\left(d_{i}^{\text{INS}}\right)}^{2}$ and ${\left(d_{i}^{\text{Re}al}\right)}^{2}$ is denoted as $\Delta d_{i}^{2}$, and it is expressed as Equation (24):

(24) $$\Delta d_{i}^{2}={\left(d_{i}^{\text{INS}}\right)}^{2}-{\left(d_{i}^{\text{Re}al}\right)}^{2}={\left(P_{E}^{\text{INS}}-x_{i}\right)}^{2}+{\left(P_{N}^{\text{INS}}-y_{i}\right)}^{2}-\left[{\left(P_{E}^{\text{Re}al}-x_{i}\right)}^{2}+{\left(P_{N}^{\text{Re}al}-y_{i}\right)}^{2}\right],i=1,2,\cdots ,m$$

The real robot position can be computed by Equation (25):

(25) $$P_{E}^{\text{Re}al}=P_{E}^{\text{INS}}-\delta P_{E},P_{N}^{\text{Re}al}=P_{N}^{\text{INS}}-\delta P_{N}$$

Thus, the Equation (24) can be transferred to Equation (26):

(26) $$h_{d_{i}}\left(\delta P_{E},\delta P_{N}\right)=\Delta d_{i}^{2}=2\left(P_{E}^{\text{INS}}-x_{i}\right)\delta P_{E}+2\left(P_{N}^{\text{INS}}-y_{i}\right)\delta P_{N}-\left(\delta P_{E}^{2}+\delta P_{N}^{2}\right),i=1,2,\cdots ,m$$

The final matrix of the measurement equation at *k* moment is illustrated in Equation (27):

(27) $$\underset{y_{k}}{\underset{︸}{\left[\begin{array}{c} \Delta V_{E,k}\\ \Delta V_{N,k}\\ \Delta d_{1,k}^{2}\\ \Delta d_{2,k}^{2}\\ \vdots \\ \Delta d_{m,k}^{2} \end{array}\right]}}=\underset{h\left(X_{k}\right)}{\underset{︸}{\left[\begin{array}{c} \delta V_{E,k}\\ \delta V_{N,k}\\ h_{d_{1}}\left(\delta P_{E,k},\delta P_{N,k}\right)\\ h_{d_{2}}\left(\delta P_{E,k},\delta P_{N,k}\right)\\ \vdots \\ h_{d_{m}}\left(\delta P_{E,k},\delta P_{N,k}\right) \end{array}\right]}}+\upsilon_{k}$$

where **υ***_k_* is the Gaussian process noise,(Δ*V_E_*, Δ*V_N_*) are the differences between INS velocity and WSN velocity in the east and north direction respectively. It is assumed that **ω***_k_* and **υ***_k_* are independent zero-mean white Gaussian sequences with covariance **Q** and **R**, respectively.

**Figure 2.**
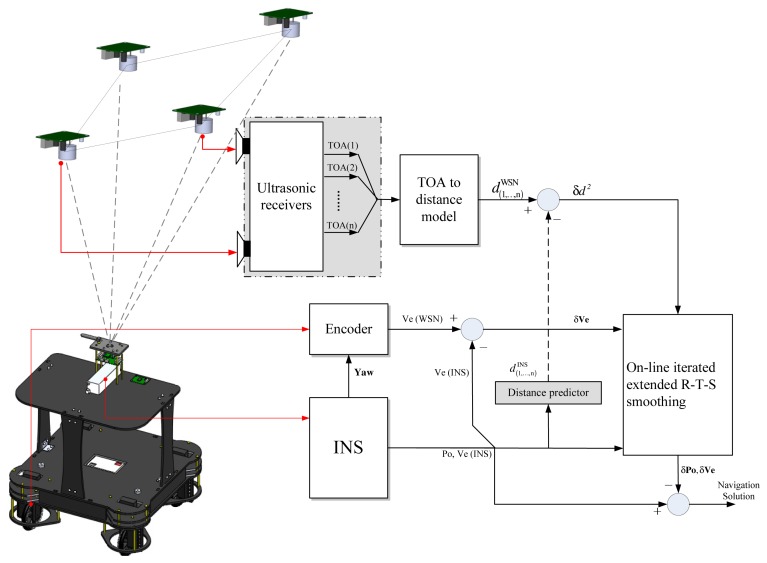
The configuration of the data fusion for the integrated navigation.

The configuration of the data fusion for the integrated navigation in this work is shown in Figure 2.

## 4. Indoor localization Tests and Performance

### 4.1. Real Indoor Test Environment

**Figure 3.**
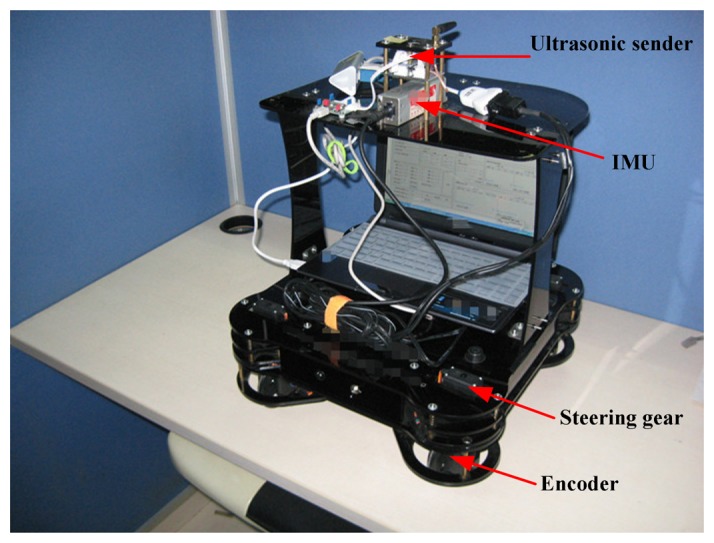
The prototype of the robot.

**Figure 4.**
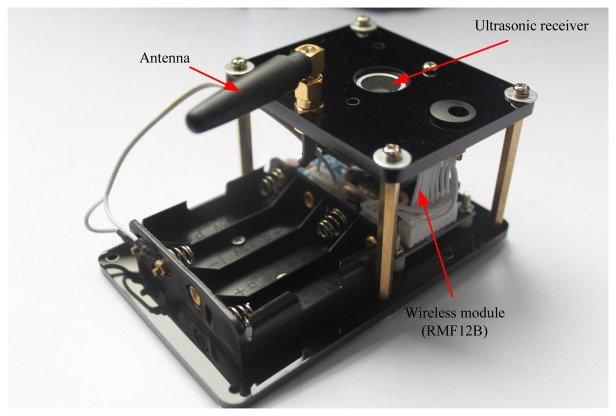
The prototype of the RN.

**Figure 5.**
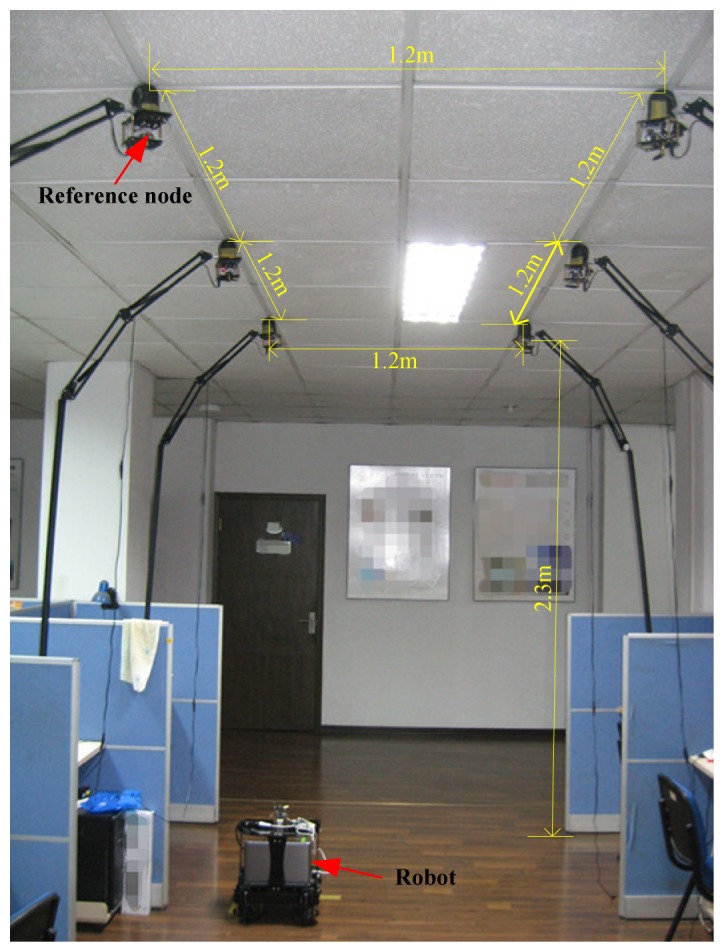
Real indoor test environment.

In this work, two real indoor tests were done to assess the performance of the proposed method. The testbed composes of one robot and six RNs. The prototype of the robot used in this work is shown in Figure 3. The robot is composed of an IMU, an ultrasonic sender and a wireless communication board. Its size is 380 mm × 380 mm × 400 mm (length × width × height). The performance characteristics of the IMU used in this work are listed in Table 1. The robot is the carrier of the IMU and the ultrasonic sender. It is able to collect the IMU datum and the distances between the robot and the RNs by using the PC fixed on the robot. Figure 4 shows the implemented prototype of the RN. Its size is 120 mm × 60 mm × 80 mm (length × width × height). Here, the RN is used to receive the ultrasonic ranging signal sent by the ultrasonic sender and the distance between the RNs and robot can be calculated. It is also able to send the sensor data to the ultrasonic sender when it gets the command. The real indoor test environment is shown in Figure 5, and the positions of the RNs are also marked in Figure 5.

**Figure 6.**
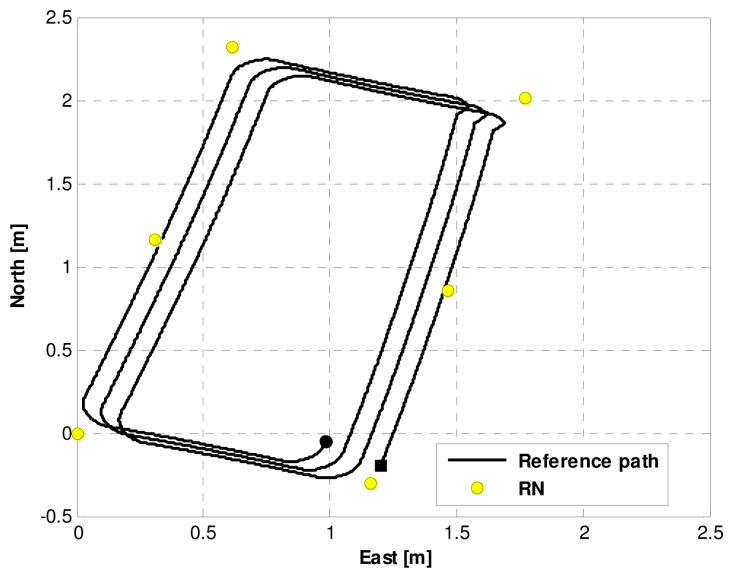
The trajectory of the real test.

Figure 6 displays the trajectory of the real test. The robot runs from the beginning point (denoted by a black square) to the end point (denoted by a black circle) at a speed of 0.33 m/s. Meanwhile, the RNs are denoted by yellow circles in Figure 6.

### 4.2. Algorithm Implementation

The pseudocode of the main program is presented in Table 2. In the pseudocode, the percentage symbol, “%,” is used to mark the comments. The software methodology is implemented in the Matlab programming language. The data refresh rate of the netbook computer is 50 Hz. Sensor data can be stored at the end of each test for subsequent analysis.

### 4.3. The Performance of the Off-Line IERTSS

**Figure 7.**
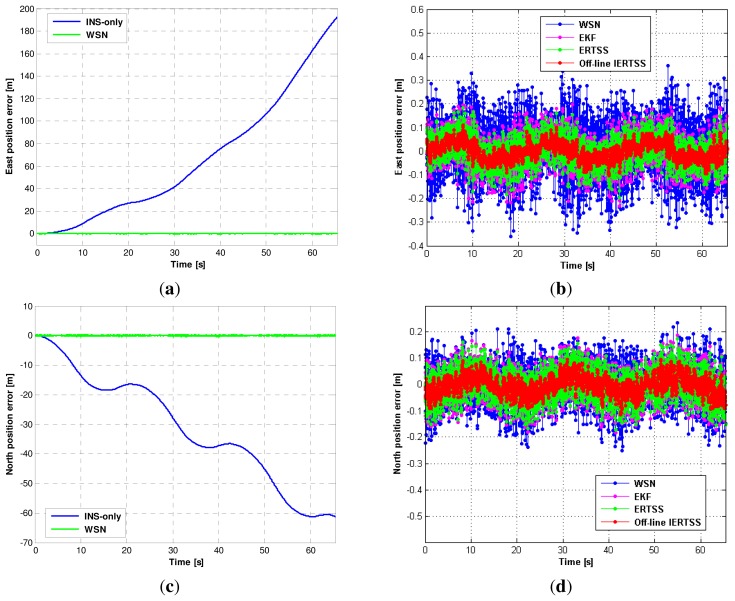
The position error for the INS-only, WSN, EKF, ERTSS and off-line IERTSS. (**a**) and (**b**) East direction; (**c**) and (**d**) North direction.

In this section, the experimental results when the off-line IERTSS works are discussed. In Figure 7, the position errors for INS-only and WSN in the east direction and north direction are shown in Figure 7a,c, respectively. The position errors for the WSN, EKF, ERTSS and off-line IERTSS in the east direction and north direction are shown in Figure 7b,d, respectively. Furthermore, the root mean square errors (RMSE) of position and velocity for the INS-only, WSN, EKF, ERTSS and off-line IERTSS are shown in Tables 3 and 4, respectively.

Figure 7a shows the east position error of the INS-only and WSN. From the figure, one can easily see that though the INS-only solution is continuous, and the east position INS error is accumulated since the DR-based current position has to depend on the previous moment. In Figure 7a, the east position INS error increases to about 190 m in 65 s without any correction. The RMSE of the INS-only solution is 85.89 m. Thus, it is necessary to correct the INS solution. Moreover, we can see that the WSN is able to maintain the east position error compared with the INS-only solution. Table 3 shows that its RMSE is 11.78 cm, which is lower than the INS solution. The east position errors for the WSN, EKF, ERTSS and off-line IERTSS are shown in Figure 7b. From the figure, it can be seen that the estimation accuracy in terms of east position for EKF is superior to that for WSN. The EKF reduces the RMSE in the east direction by about 41.17% compared with the WSN solution. Regarding the smoothing methods, it is evident that both the ERTSS and the off-line IERTSS are effective to reduce the RMSE. Table 3 shows that the position RMSE for the off-line IERTSS is lower than that for ERTSS. The off-line IERTSS reduces the position RMSE by about 38.22% compared with ERTSS.

The north position errors for the INS-only, WSN, EKF, ERTSS and off-line IERTSS are shown in Figure 7c,d, respectively. The trend in this figure is similar to that in Figure 7a,b. The north position error of INS is also accumulated. The WSN solution is also able to maintain the accuracy of the position. From Table 3, we can see that the RMSE of the north position for WSN remains at about 7.71 cm since the WSN solution just depends on the current measurement. Like Figure 7b, the off-line IERTSS solution also has the lowest error. The RMSE of the north position for the off-line IERTSS is 3.46 cm. The improvement in RMSE is about 43.37% and 37.66% compared with the EKF and ERTSS, respectively. In summary, the off-line IERTSS is the most effective method to reduce the position error compared with the INS-only, WSN, EKF and ERTSS.

The RMSE results of velocity for the INS-only, WSN, EKF, ERTSS and off-line IERTSS are shown in Table 4. From the table, it can be seen that the off-line smoothing-based methods are able to effectively reduce the velocity error of the filter. However, the ERTSS and off-line IERTSS solution are almost the same both in the east and north direction, respectively.

### 4.4. The Relationship between Smoothing Window Size and Accuracy for the On-Line IERTSS

**Figure 8.**
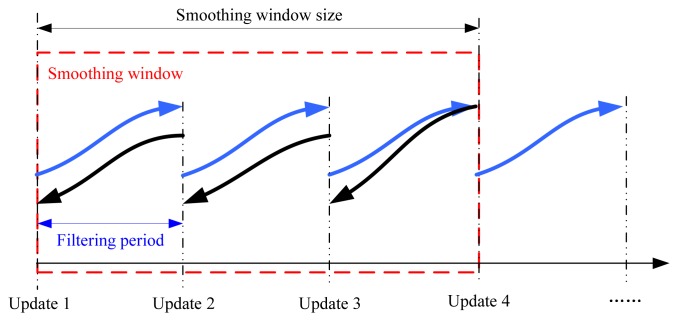
The relation between the smoothing window size and the filtering period.

In this section, the relationship between smoothing window size and accuracy for the on-line IERTSS is discussed. The relation between the smoothing window size and the filtering period is shown in Figure 8. From the figure, we can see that the relation can be expressed by the following equation:

(28) (Smoothing window size)=n⋅(Filtering period)=n⋅T

**Figure 9.**
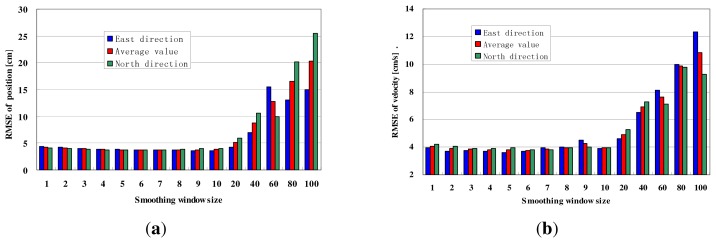
Relationship between smoothing window size and RMSE. (**a**) Position and (**b**) Velocity.

Tables 5 and 6 and Figure 9 show the testing results. From the results, we can see that both the RMSE of position and that of velocity reduce as the smoothing window size increases on the beginning, then the RMSE increases rapidly.

From Table 5, we can see that RMSE of position for the on-line IERTSS is lowest when the smoothing window size is 6, so for the velocity, the solution is 6. Table 5 displays the RMSE of position in the east and north direction with the different smoothing window sizes, while, the average position RMSE in the east and north direction is also shown in the last line. The position RMSE in the east and north direction with the different smoothing window size is shown in Table 6, and the average position RMSE in the east and north direction is also shown in the last line. In order to obtain a high accuracy navigation solution, we take the average of the average RMSE for position and velocity, and the result is shown in Table 7. From that table, we can see when the smoothing window size is 6, the average value is lowest.

### 4.5. Comparison of On-Line and Off-Line IERTSS

**Figure 10.**
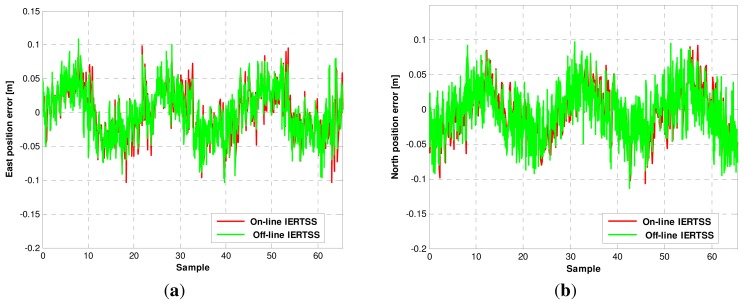
The position error for the off-line IERTSS and on-line IERTSS. (**a**) East direction and (**b**) North direction.

In this section, the comparison of on-line and off-line IERTSS is discussed. The position errors for the off-line and on-line IERTSS are shown in Figure 10. The comparison of on-line and off-line mode in terms of position error is shown in Table 8. From the figures, one can see that both in the east direction and north direction, the position error of the on-line and the off-line IERTSS solutions are almost the same, and the off-line mode is a little better than the on-line mode. Table 9 displays the comparison of on-line and off-line mode in terms of velocity error. Like the position error, the on-line and the off-line IERTSS solutions are also almost the same, and the off-line mode is better than the on-line mode. However, the performance of on-line IERTSS is little better than the ERTSS.

## 5. Conclusions

This work proposed an on-line IERTSS for tightly integrated INS/WSN mobile robot navigation indoors. In this mode, IEKF is employed instead of the EKF in forward data processing of the ERTSS. Then, IERTSS is embedded into average filter for on-line smoothing. The experimental results show that the proposed on-line smoothing outperforms the ERTSS. The performance of on-line smoothing is also comparable to that of off-line smoothing. The results show that the performance of the on-line and off-line mode is almost the same, and the off-line mode is a little better than the on-line mode.

## Figures and Tables

**Table 1.** The performance parameters for the IMU used in this work.

**Parameters**	**Description**	**Data**
Angular Rate	Input Range: Yaw, Pitch, Roll	±300 deg/s
	Bias	0.02 deg/s RMS
	Scale Factor Accuracy	0.2%
	Non-Linearity	0.1% FS
	Random Walk	6 deg/sqrt (h)
Linear acceleration	Input Range: X/Y/Z	±2 g
	Bias	0.3 mg RMS
	Scale Factor Accuracy	<0.1%
	Non-Linearity	0.2% FS
	Random Walk	0.06 deg/sqrt (h)

**Table 2.** The pseudocode of the proposed algorithm.

**State (s), Covariance (Cov), Position (Po), Velocity (Ve), Probability (PP)**	
1.	**procedure** MAIN % Main program
2.	[**_S, Cov, Po, Ve, PP_**] ← Initialize();
3.	Start IMU & ultrasonic measurements ();
4.	**loop** % 50 Hz rate
5.	WaitNextIMUSample;
6.	[**ω***^b^*,**f***^b^*,**Yaω**] ← GetIMUData();
7.	[ $V_{E}^{\text{INS}}$, $V_{N}^{\text{INS}}$] ← IMUattitudesolution(**ω***^b^*,**f***^b^*);
8.	[ $P_{E}^{\text{INS}}$, $P_{N}^{\text{INS}}$] ← DeadReckoning( $V_{E}^{\text{INS}}$, $V_{N}^{\text{INS}}$);
9.	Data_Ultrasonic_ ← GetUltrasonicData();
10.	$TOA_{\left(1,\ldots ,n\right)}^{\text{Ultrasonic}}$ ← MeanTOA(Data_Ultrasonic_);
11.	$d_{\left(1,\ldots ,n\right)}^{\text{WSN}}$ ← TOAtoDistanceModel( $TOA_{\left(1,\ldots ,n\right)}^{\text{Ultrasonic}}$);
12.	$d_{\left(1,\ldots ,n\right)}^{\text{INS}}$ ← INSPositionToDistanceModel( $P_{E}^{\text{INS}}$, $P_{N}^{\text{INS}}$);
13.	$V_{e}^{\text{WSN}}$ ← GetVelFromCodeWheel();
14.	[ $V_{E}^{\text{WSN}}$, $V_{N}^{\text{WSN}}$] ←GetNorth&EastVel( $V_{e}^{\text{WSN}}$, **Yaω**);
15.	**z**← [**δVe, δd^2^**]; % Measurements
16.	**Cov**_z_ ← GetCovZ(**z**);
17.	**S**← [ $Po_{i-1}^{+}$, $Ve_{i-1}^{+}$];
18.	**Cov** ← [ $PP_{i-1}^{+}$, $PP_{i}^{-}$, **Cov***_z_*];
19.	[**S,Cov**] ← **IEKF**(**z,S,Cov**);
21.	FilterData ← StoreFilterData(**S,Cov**);
22.	if (OutputData = = 1)
23.	[**δP***_E_***,δP***_N_***,δP***_E_***,δP***_N_*]_IERTSS_ ← IERTSS(**S,Cov**);
24.	[**P***_E_***,P***_N_***,V***_E_***,V***_N_*]_IERTSS_ ←[ $P_{E}^{\text{INS}}$, $P_{N}^{\text{INS}}$, $V_{E}^{\text{INS}}$, $V_{N}^{\text{INS}}$] –[**δP***_E_***, δP***_N_***,δP***_E_***,δP***_N_*]_IERTSS_
25.	[**P***_E_***,P***_N_***,V***_E_***,V***_N_*]_Avg. IERTSS_ ←GetAverageValue([**P***_E_***,P***_N_***,V***_E_***,V***_N_*]_IERTSS_);
26.	OutputData();
27.	endif
28.	**end loop**
29.	Stop IMU & ultrasonic measurements ();
30.	StoreSession(All variables); % For ananlysis
31.	**end procedure**

**Table 3.** Comparison of five estimation strategies in terms of position error.

**Method**	**RMSE (cm)**		

	**East**	**North**	**Mean**
INS-only	8589.14	3625.92	6107.53
WSN	11.78	7.71	9.74
EKF	6.93	6.11	6.52
ERTSS	5.73	5.55	5.64
Off-line IERTSS	3.54	3.46	3.50

**Table 4.** Comparison of five estimation strategies in terms of velocity error.

**Method**	**RMSE (cm/s)**		

	**East**	**North**	**Mean**
INS-only	343.49	131.48	237.48
WSN	5.73	8.79	7.26
EKF	4.07	6.12	5.10
ERTSS	3.15	4.73	3.94
Off-line IERTSS	2.77	2.79	2.78

**Table 5.** Relationship between smoothing window size and position RMSE (cm).

**Size**	**East**	**North**	**Average**
1 T	4.37	4.16	4.27
2 T	4.22	3.95	4.09
3 T	4.07	3.84	3.96
4 T	3.94	3.73	3.84
5 T	3.83	3.75	3.79
6 T	3.72	3.73	3.73
7 T	3.71	3.77	3.74
8 T	3.69	3.83	3.76
9 T	3.65	3.97	3.81
10 T	3.60	4.06	3.83
20 T	4.30	5.92	5.11
40 T	7.02	10.59	8.80
60 T	15.56	10.01	12.78
80 T	13.02	20.21	16.61
100 T	15.06	25.47	20.26

**Table 6.** Relationship between smoothing step and velocity RMSE (cm/s).

**Size**	**East**	**North**	**Average**
1 T	3.94	4.21	4.07
2 T	3.72	4.07	3.89
3 T	3.78	3.92	3.85
4 T	3.71	3.90	3.81
5 T	3.61	3.98	3.79
6 T	3.68	3.80	3.74
7 T	3.93	3.81	3.87
8 T	4.02	3.94	3.98
9 T	4.52	3.98	4.25
10 T	3.90	3.97	3.93
20 T	4.59	5.24	4.92
40 T	6.52	7.27	6.90
60 T	8.10	7.11	7.61
80 T	9.98	9.76	9.87
100 T	12.35	9.30	10.83

**Table 7.** The average of average RMSEs for position and velocity.

**Size**	**Position (cm)**	**Velocity (cm/s)**	**Average**
1 T	4.27	4.07	4.17
2 T	4.09	3.89	3.99
3 T	3.96	3.85	3.90
4 T	3.84	3.81	3.82
5 T	3.79	3.79	3.79
6 T	3.73	3.74	3.73
7 T	3.74	3.87	3.81
8 T	3.76	3.98	3.87
9 T	3.81	4.25	4.03
10 T	3.83	3.93	3.88
20 T	5.11	4.92	5.01
40 T	8.80	6.90	7.85
60 T	12.78	7.61	10.19
80 T	16.61	9.87	13.24
100 T	20.26	10.83	15.55

**Table 8.** Comparison of on-line and off-line mode in terms of position error.

**Method**	**RMSE (cm)**		

	**East**	**North**	**Mean**
Off-line IERTSS	3.54	3.46	3.50
On-line IERTSS	3.72	3.73	3.73

**Table 9.** Comparison of on-line and off-line mode in terms of velocity error.

**Method**	**RMSE (cm/s)**		

	**East**	**North**	**Mean**
Off-line IERTSS	2.77	2.79	2.78
On-line IERTSS	3.68	3.80	3.74
